# A Hirschsprung Pull-through, “with a Twist”

**DOI:** 10.1055/s-0040-1717128

**Published:** 2021-01-09

**Authors:** Hira Ahmad, Devin R. Halleran, Raquel Quintanilla, Alessandra C. Gasior, Richard J. Wood, Marc A. Levitt

**Affiliations:** 1 Department of Pediatric Colorectal and Pelvic Reconstructive Surgery, Nationwide Children's Hospital, Columbus, Ohio, United States

**Keywords:** Hirschsprung disease, pull-through reoperation, twisted pull-through, enterocolitis, congenital intestinal obstruction

## Abstract

Hirschsprung disease is the most common neurocristopathy in children, resulting in the congenital loss of enteric ganglia. Surgery, which involves resecting the aganglionic segment and restoring bowel continuity, usually results in a good outcome; however, some patients suffer from multiple episodes of enterocolitis and other obstructive symptoms. A contrast enema, examination under anesthesia, and rectal biopsy can identify the cause of obstruction in many cases, including a rare explanation, a twist of the pull-through, a case of which we present here.

## Background

Hirschsprung disease (HD) is a congenital intestinal obstruction due to the absence of ganglion cells in the myenteric and submucosal plexuses of the distal intestine, with a one in 5,000 live-born incidence. 1 Its management involves resection of the aganglionic bowel and pull-through of normal bowel. 2 Most patients do well after surgery; however, some patients struggle with obstructive symptoms and recurrent episodes of enterocolitis. 3 The common causes for such symptoms include an anastomotic stricture, a retained obstructing Soave cuff, a large Duhamel pouch, a transition zone or a retained Hirschsprung segment, and in some rare cases, a twisted pull-through. 4 Most of these problems can be identified by a contrast study, examination under anesthesia, and/or a repeat rectal biopsy. A twisted pull-through is difficult to identify on a contrast study or examination. We present such a case and describe subtle cues on contrast study and rectal exam that will be indicative of a twisted pull-through.

## Presentation

A 5-year-old male underwent a transanal laparoscopic assisted Soave pull-through as a newborn. At 1 year of age, he presented with daily obstructive symptoms of distention and bloating. He suffered from three episodes of enterocolitis, each requiring hospital admission. At an outside hospital, he underwent an examination under anesthesia and a full thickness rectal biopsy with findings of an intact dentate line, no anastomotic stricture, and a palpable Soave cuff on the anterior rectal wall only. His rectal biopsy showed ganglion cells, normal size nerve fibers, normal anticholinesterase reaction, a normal calretinin staining pattern, and his contrast study was felt to be normal. The patient also underwent colonic manometry testing which showed diffuse colonic dysmotility. Based on this result, an ileostomy was recommended.

Thereafter, the patient thrived. The patient then returned for a planned repeat manometry study 6 months later which now showed normal motility, and the recommendation was to close the ileostomy. Prior to closing the ileostomy, the family sought a second opinion.

**Fig. 1:**
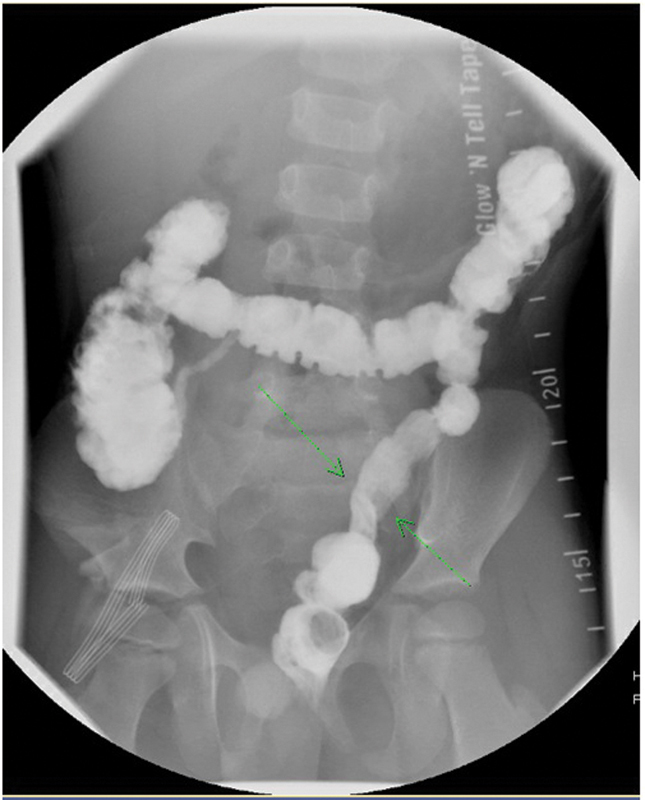
Postevacuation film showing a twist of the pull-through demonstrated by a shift in the mesenteric border of the pull through segment.

**Fig. 2:**
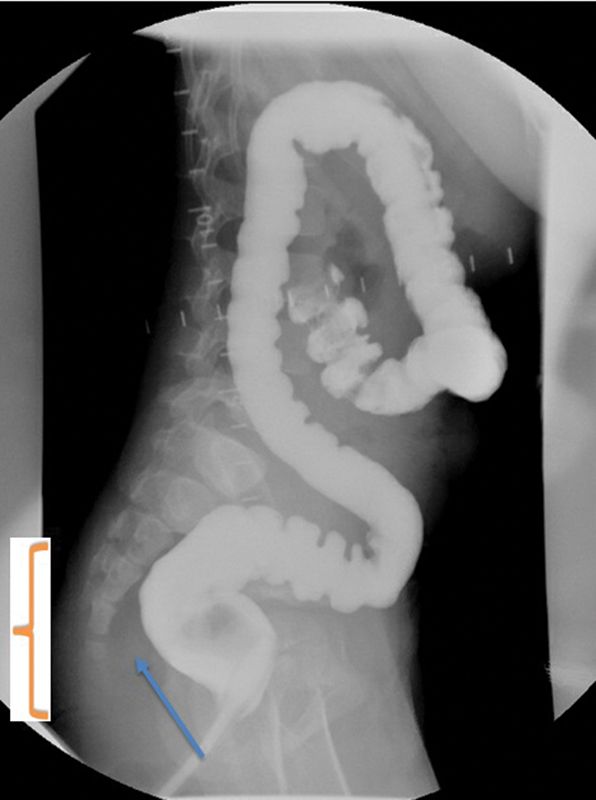
Contrast enema with absence of haustrations in the distal segment denoted by “}.” The arrow denotes widening of the presacral space.

**Fig. 3:**
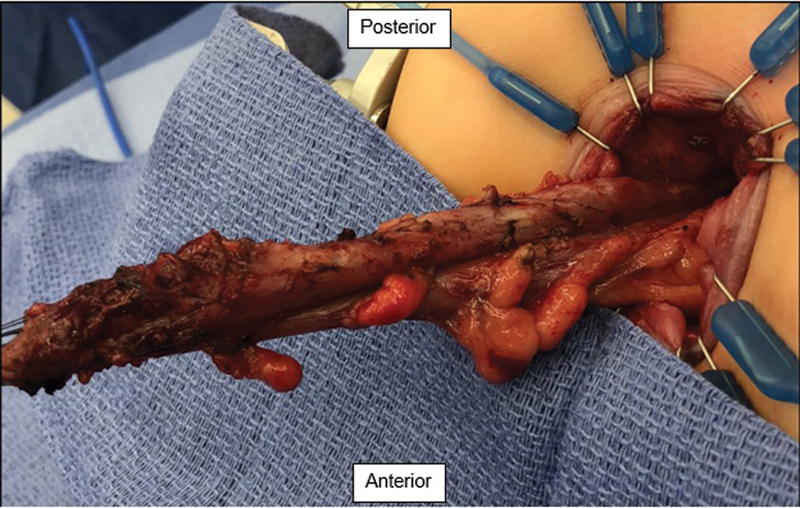
Twist in the pull-through segment with the mesentery noted on the anterior aspect (the patient is in prone position).

Our review of the post pull-through contrast enema ( Fig. 1 ) came to a different conclusion. We felt the study showed a 180-degree twist in the distal pull-through ( Fig. 1 ). This was demonstrated by the shift in the antimesenteric border of the colon. We also felt that the twist was intermittent since the contrast study did not demonstrate bowel dilatation proximal to the twist. Since this patient had a previous Soave pull-through, it was also important to note the presence of presacral space widening ( Fig. 2 ), which could be indicative of a Soave cuff; however, this was not evident on the physical exam. Our repeat examination under anesthesia and rectal biopsy confirmed the findings from the outside hospital of no circumferential Soave cuff; however, we were unable to pass a digit or a Foley catheter freely into the pelvis. Given the recurrent obstructive symptoms that occurred prior to the ileostomy and the concerns on the contrast study and exam, we offered the patient a reoperation. We performed a Swenson type redo transanal only pull-through and upon transanal mobilization the distal pull-through was noted to have a 180-degree twist, with the mesentery on the anterior side when it should have been either medial or posterior ( Fig. 3 ). We untwisted the pull-through and redid the coloanal anastomosis and confirmed no twist by freely passing the foley catheter into the pelvis. The patient did well postoperatively and was discharged home on postoperative day 4. He was seen for a follow-up at 1 month, which showed a well-healed pull-through with no anastomotic stricture and his ileostomy was closed. He continues to thrive with no distention or bloating, and stools one to two times daily with control, now 1 year after his ileostomy closure.

## Discussion

The most common complications reported after surgery for HD are constipation, fecal incontinence, and enterocolitis. 3 These can have a considerable effect on the child's overall well-being. Failure to thrive and obstructive symptoms post pull-through are usually secondary to anatomical or pathological problems. 5 6 An evaluation including history and physical examination, a contrast enema, examination of the anus and pull-through under anesthesia, and a full thickness rectal biopsy are required to assess a patient with obstructive problems post pull-through. 5 7 Rectal biopsy rules out a pull-through done in an aganglionic segment or a pull-through of the transition zone. Contrast enema and examination of the anus assesses for an anastomotic stricture, a retained Soave cuff, a retained dilated segment of colon, a mega Duhamel pouch or a twist or kink in the pull-through segment. 1 3 Often with no anatomic or pathologic explanation, nonrelaxing sphincters are the culprit and can be treated with botulinum toxin. 1

In the patient presented here, severe obstructive symptoms led to a diverting ileostomy because of the finding of diffuse colonic dysmotility. We question such a manometry finding in the presence of a distal obstruction and in our practice do not do this study until all possible causes of obstruction are ruled out. 4 5 At the prior hospital, no obstructive causes were found which may have led to the decision to do colonic manometry. Then, after the ileostomy, when he returned and had “normal” motility on the repeat study, the recommendation was to close his ileostomy. But, had that been done, we predict similar obstructive issues would likely have occurred.

Manometry has been used in the evaluation of the motility of postoperative HD patients because it has been shown that constipation and bowel distention may occur as a result of the absence of high-propagating contractions, 8 a concept extrapolated from patients with functional constipation. High-anal resting pressure (a key characteristic of all HD patients' sphincters) and a weak neorectal peristalsis may cause constipation, while poor neorectal compliance and elevated neorectal pressures in the presence of normal or low anal resting pressure can cause fecal incontinence, 9 the latter potentially related to iatrogenic overstretching of the anal sphincters at the time of the pull-through. 10

In this case, we found a twist in the pull-through segment. This is an extremely rare complication with the incidence reported in the literature to be less than 1%. 11 The twist was suspected on the contrast study and on digital exam. It was clearly seen after the pull-through was dissected, with the mesentery on the anterior side. The clues to a twist include obstructive symptoms without an obvious distal obstruction (secondary to Soave cuff or aganglionic pull-through). Often there is no proximal dilation because the twist causes intermittent obstruction. It needs to be suspected and seen on contrast enema or felt on rectal exam. 12

Twisting of the proximal bowel can be avoided by paying careful attention to the orientation of the mesenteric blood supply to the pull-through segment. Intraoperatively, we frequently pass a Foley catheter before and after completing the anastomosis to ensure smooth passage and the pull-through segment is not kinked. Other institutions have also reported performing a colonoscopy to check for twist. After redo and subsequent ileostomy closure, the patient presented here had no further obstructive symptoms.

## Conclusion

For patients with obstructive symptoms post pull-through, a thorough and systematic workup is warranted to rule out an anatomical or pathological reason for this constellation of symptoms. 7 Careful inspection of the contrast study and a thorough digital examination under anesthesia must be performed to check for a twist. If an anatomical problem is found, a redo operation can significantly improve the quality of life in these patients.

## Notes

New Insights and the Importance for the Pediatric Surgeon Careful inspection of the contrast study and a thorough examination under anesthesia must be performed to check for a twist in patients with obstructive symptoms post pull-through.
